# Navigating the sea level rise: Exploring the interplay of climate change, sea level rise, and coastal communities in india

**DOI:** 10.1007/s10661-024-13191-z

**Published:** 2024-10-03

**Authors:** Ansuman Das, Pranaya Kumar Swain

**Affiliations:** School of Humanities and Social Sciences, National Institute of Science Education and Research, An OCC of Homi Bhabha National Institute, Bhubaneswar, 752050 Odisha India

**Keywords:** Sea level rise, Climate change, Coastal livelihoods, Socio-economic impacts, Sustainable development, Biodiversity conservation

## Abstract

This research article investigates the intricate interplay between climate change, global sea level rise (SLR), and the impacts of sea level rise on the coastal regions of India. Through an interdisciplinary approach, this paper provides an overview of the global consequences of SLR on coastal communities, exploring economic, social, and environmental impacts on agriculture, communities, and coastal areas. The study examines the displacement of communities and its impact on food security, infrastructure, tourism, and ecological loss based on a comprehensive literature review. This paper emphasizes the sustainable preservation of coastal ecosystems and the development of climate-resilient infrastructure. This research aims to offer a detailed understanding of the evolving landscape of coastal livelihoods, providing valuable insights for adaptive strategies, policy formulation, and sustainable development. Ultimately, this article contributes to the scientific discourse by shedding light on the complex dynamics between climate change, SLR, and coastal communities, guiding efforts toward a resilient and sustainable future. The insights are drawn from secondary data resources, including books, scholarly journals, and reports from organizations such as the IPCC and NOAA. Based on a thorough review of the relevant literature, it critically examines the existing and potential consequences of sea level rise induced by climate change.

## Introduction

Proximity to the coast and altitude are important geographical factors that influence where people choose to settle. Being close to the sea is important as it provides benefits such as trade, livelihood options, and access to resources (Kummu et al., 2016; McGaugh, 1970). Coastal areas are important because of their high population density, substantial investment in built infrastructure, and the presence of valuable ecological resources (NOAA, 2013). Throughout history, living near the coast has been appealing to both individuals and businesses due to benefits like trade, livelihood options, and access to resources. Today, millions depend on coastal proximity for their livelihoods (Brown et al., 2014). Global warming is making sea levels rise in two ways: ice melting adds water to the ocean, and the warming water expands, taking up more space (NOAA, 2021). Rising sea levels are placing pressure on coastal areas, affecting their role in providing recreation, storm protection, and home to diverse marine life, including vital fisheries. Additionally, rising seas are polluting underground freshwater sources, which are essential for towns, farms, and natural environments (Lindsey, 2022). Sea levels are rising as a result of glaciers melting and oceans warming due to climate change. Since 1880, global sea levels have risen by almost eight inches, and experts predict it could rise by another 1 to 4 ft by the year 2100 (Church and White, 2011). The increase began in the twentieth century and is likely to accelerate due to human-induced warming in the twenty-first century. According to the Third Assessment Report (TAR) of the Intergovernmental Panel on Climate Change (IPCC), the projected sea level rise from 1990 to 2100 ranged from 9 to 88 cm, with a median estimate of 48 cm (Church and White, 2011). Sea level rise (SLR) could be one of the most expensive and lasting impacts of climate change. According to a 2001 report by the Intergovernmental Panel on Climate Change (IPCC), the coastal environment, where land meets the sea, is a dynamic and important region that supports a variety of productive habitats (Hauer et al., 2020). Issues such as sea level rise and climate change are causing significant challenges to coastal habitats and human health, such as deforestation, fertilizer runoff from agricultural lands, and untreated sewage (Barbier et al., 2011). In 2002, the United Nations Environment Programme (UNEP) warned that about 40% of the human population lives within 60 km of the coast globally, and pollution in these areas is causing a serious health crisis. The natural processes that shape coastal zones and their ecosystems are vital, but human activities are threatening them (Crossland et al., 2005). Nearly half the people in the United States, about 52% (a total of 163.8 million people in 2010), live in areas close to the ocean, the Gulf of Mexico, or the Great Lakes, or in places at risk of flooding. The population density in these areas is three to four times higher than in other parts of the country. Additionally, compared to states away from the coast, states near the coast have more elderly people who may be vulnerable in an emergency (NOAA, 2013). India, with its extensive coastline spanning between 8°N and 37°N latitudes, experiences significant impacts of climate change. The country’s peninsular shape and surrounding water bodies, including the Bay of Bengal to the east, the Indian Ocean to the south, and the Arabian Sea to the west, cover approximately 7500 km of coastline. It plays an important role in influencing factors such as rainfall, monsoon winds, air currents, and tropical cyclones (Panda, 2020; Wilson et al., 2020). The reports by Unnikrishnan et al. (2006) and Unnikrishnan and Shankar (2007) forecast a notable rise in temperature of 4.4 °C by the end of the current century. They also point out evidences of sea level rise along India’s east coast, highlighting the persistent effects of climate change on the country’s weather patterns and coastal regions, particularly regarding increasing sea levels.

## Objectives

The primary objective of this review is to examine the historical trends of sea level rise at the global scale with special emphasis on coastal areas in India. Additionally, the study will review climate change impact studies focusing on coastal communities in India. Through these interconnected objectives, the study aims to provide a holistic understanding of the strategies employed for adaptation and mitigation of climate change–induced sea level rise.

## Methodology

This study draws insights from secondary data sources, including books, scholarly journals, published papers, and official reports from the Intergovernmental Panel on Climate Change (IPCC) and the National Oceanic and Atmospheric Administration (NOAA). Data were collected from key reports such as Church and White’s (2011) study, “Sea-Level Rise from the Late 19th to the Early 21st Century,” which provided datasets on historical and projected global sea level rise, and Dasgupta et al.’s (2009) report, “The Impact of Sea Level Rise on Developing Countries: A Comparative Analysis,” which offered data on sea level rise scenarios and their impacts, particularly in India. The analysis reflects upon examining historical trends using Church and White’s data to gain an insightful understanding of long-term sea level changes and comparing these with future projections from Dasgupta et al. to assess potential impacts on coastal areas. The findings were synthesized to draw conclusions about the current and future effects of sea level rise, with a particular focus on India.

## Theoretical underpinning

Sociological theory associated with the study of climate change is the “Risk Society” theory proposed by German sociologist Ulrich Beck. The Risk Society theory suggests that modern societies are characterized by a shift from industrial to post-industrial forms of organization, and with this shift comes a new set of risks and uncertainties. Beck argues that industrial societies have created new technologies and systems that simultaneously produce benefits and risks, with environmental risks, including those related to climate change, being prominent examples (Jong, 2022). In the context of climate change, the Risk Society theory emphasizes how the industrial activities that have driven economic development also generate environmental risks that are global in scale. Beck further argues that traditional institutions and ways of thinking are ill-equipped to manage these global risks effectively. Climate change, with its potential for widespread and unpredictable consequences, exemplifies the types of risks that the theory addresses. Ulrich Beck’s Risk Society theory is of immense relevance to the issue of sea level rise in India, as it highlights how global risks impact local communities. Beck’s theory underscores that while sea level rise is a global environmental concern driven by climate change, its effects are intensely local, particularly for India’s coastal areas where rising seas threaten infrastructure, livelihoods, biodiversity, and ecosystems. Beck’s theory further helps in understanding public risk perception and awareness, as well as the role of globalization in shaping local responses through transnational cooperation and knowledge sharing. Overall, Beck’s theory provides a comprehensive lens through which we can analyze and address the complex challenges posed by sea level rise in India.

## Climate change: The driving force behind rising sea levels

Human activities, especially the burning of fossil fuels, are causing rapid warming, leading to serious consequences such as drought, water scarcity, fires, sea level rise, floods, melting of polar ice caps, storms, and loss of biodiversity. By 2030–2050, climate change is projected to result in approximately 250,000 additional deaths annually, primarily due to malnutrition, malaria, diarrhea, and heat stress (WHO, 2023). Since 1900, the Earth’s average surface temperature has risen by approximately 1 °C (1.8 °F). Climate change, a serious challenge of our times, is now clearly linked to human activities. A warming atmosphere and oceans are causing sea levels to rise, Arctic sea ice to decline significantly, and many other climate-related changes, underscoring the urgency of addressing this global issue (Sciences & U.A 2014). The average temperature of Earth’s land and oceans has increased by about 2 °F (1.1 °C) since 1850, according to NOAA’s 2023 annual climate report. Over the past few decades, since 1982, the rate of temperature increase has been more than three times faster, with an increase of about 0.36 °F (0.20 °C) per decade (NOAA, 2024). Sea levels are rising in coastal areas due to global warming. From 1901 to 2018, the global average sea level rose by 0.20 m. It grew at a rate of 1.3 mm per year from 1901 to 1971, then increased to 1.9 mm per year from 1971 to 2006. Now, the rise has accelerated even further, at a rate of 3.7 mm per year between 2006 and 2018, becoming a major concern for the coastal region (IPCC, 2021). Visible climatic effects, like as temperature fluctuations, changed precipitation, sea level rise, and unpredictable weather events, are being caused by greenhouse gases (GHGs) and pollutants in the atmosphere. These effects are especially noticeable in India, a country of about 1.4 billion people, where 420 million of them live along or close to the ocean (Subramanian et al., 2023).

## Historical trends in climate change and global sea level rise

The world has a total coastline of 35,6000 km, covering over 10% of the Earth’s surface. Coastal areas are vital for economic benefits like ocean navigation, fisheries, tourism, and industrialization. As a result, human settlements are concentrated along the coast, with about 40% of the global population living within 100 km of it. However, approximately 10% of the world’s population resides in low-lying coastal zones (< 10 m), making them highly vulnerable to coastal disasters. With climate change, sea levels will likely continue to rise for centuries, posing a significant risk even if we stabilize greenhouse gas emissions in the near future (Church et al., 2013). According to the prevailing scientific consensus, it is anticipated that the sea level will rise by 1 m or more by the year 2100 (Hauer et al., 2020, NOAA, 2017).

**Fig. 1:**
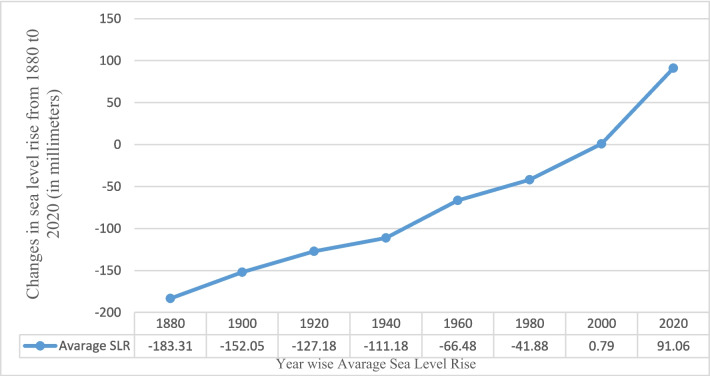
Global sea level rise. (Source: Sea level estimates from Church and White (2011). NOAA Climate.gov (2022) image based on analysis and data from Philip Thompson, University of Hawaii Sea Level Center)

Figure 1 shows how the sea level has gone up from 1880 to 2020. In 2020, it increased to 91.6 mm. These values represent the change in sea level compared to the average from 1993 to 2008 (Church & White, 2011). Since 1880, the global average sea level has gone up by 8–9 inches. In 2022, it reached a new record high—101.2 mm (4 inches) above the levels in 1993. Between 2006 and 2015, the global ocean level went up by 0.14 inches per year, which is faster than it used to be. In the twentieth century, it increased by 0.06 inches per year. By the end of this century, even if we reduce greenhouse gas emissions, the sea level is expected to go up by at least one foot above the levels in 2100 (Lindsey, 2022).

US coastal counties are facing permanent flooding and flood threats due to rising sea levels, intense rainfall, high tide flooding, and more severe storms. Additionally, it is likely that hurricane intensity has increased over the past four decades. Scientists are projecting a future scenario where a warming climate will not only lead to more powerful storms but also increase overall rainfall (IPCC, 2021). Although the exact details of sea level rise remain uncertain, scientists are confident that it will significantly impact coastal communities, even under conservative estimates. Additionally, coastal areas face other climate threats such as ocean acidification, migration, loss of livelihoods, harmful algal blooms, and saltwater intrusion (Fleming, et al., 2018).

Changes in global sea level are affected by changes in liquid water stored on land. It involves the extraction of groundwater and storage of freshwater through dam construction (Chao et al., 2008; Fiedler & Conrad, 2010). In the early 1900s, ice covered more land, causing sea levels to drop. Human activities have changed the movement of water on land like never before. However, in recent years, using too much water for homes, farms, and industry is causing sea levels to rise (Wada et al., 2017). This relationship between temperature and sea level is not new. If we look back over the last 1500 years we can see that changes in global average temperature are linked to changes in sea level. So, when the Earth warms, it can cause sea levels to rise, and when it cools, it can cause sea levels to lower. This is a relationship that highlights the impact of temperature change on our oceans in the long term (Kopp et al., 2016). Sea level rises due to melting ice and warming water. Studies show that in recent decades, more than 90% of the excess heat in our climate system has been absorbed by the ocean. This warming water helps us understand how our climate responds to changes, showing the connection between rising sea levels and the sensitivity of our climate (Church et al., 2013).

Coastal ecosystems are dealing with the challenges of rising sea levels, climate-related ocean changes, and human activities. It is difficult to pinpoint the exact impact of sea level rise due to other factors such as infrastructure development and habitat degradation. The consequences include permanent land submergence, more frequent and intense flooding, coastal erosion, ecosystem loss, soil and water salinization, and drainage issues. Looking ahead, without adaptation, most low-lying areas face substantial risk from these threats, regardless of their level of development. Effective adaptation strategies are important to mitigate these multidimensional challenges and protect coastal areas (Oppenheimer, et al., 2019).

Sea levels will increase as a result of greenhouse gas emissions causing the Earth to warm. The amount and speed depend on emission rates and potential ice sheet collapse. The 2022 report suggests that even with low emissions (1.5 °C warming), sea levels will rise by at least 0.3 m by 2100. Higher emissions, causing faster ice sheet collapse, could lead to an increase of 2 m by 2100. Reducing emissions is key to reducing sea level rise (Sweet et al., 2022).

## Climate change and sea level rise: A concern for indian coastal areas

India’s extensive coastlines on both sides face substantial impacts from climate-related events. This complex relationship between human habitats, coastal ecosystems, and climate challenges highlights the urgent need for comprehensive strategies to address the consequences of increasing greenhouse gases and pollutants (Subramanian et al., 2023). India’s coastline extends from 7° to 24° North latitude and 70° to 94° East longitude, bordered by the Arabian Sea in the west and the Bay of Bengal in the east. The total coastline is 7500 km, of which 5400 km is on the mainland and the rest is distributed over the Lakshadweep group in the Arabian Sea and the Andaman and Nicobar Islands in the eastern Bay of Bengal. About 250 million people live within 50 km of the Indian coast (Roy et al., 2023). The Government of India, through the Ministry of Earth Sciences (MoES), considers climate change as a top concern. In 2020, they released a report titled “Climate Change Assessment in the Indian Region.” A 2020 report showed that India’s average temperature rose by 0.7 °C between 1901 and 2018. The report predicts a more significant increase of 4.4 °C by the end of the twenty-first century. Summer monsoon rainfall has decreased by 6% since 1951 and is expected to increase further. The report also highlights signs of sea level rise off the east coast of India. This information emphasizes the ongoing impact of climate change on India’s weather patterns and coastal areas (Krishnan et al., 2020). Even though there are not many long-term tide-gauge stations on the Indian coasts, it is still clear that sea levels are rising. We may see significant erosion, especially in critical areas like the delta on the east coast of India. Although people initially thought that this erosion was primarily caused by human activities, it is now becoming clear that rising sea levels also play a significant role (Rao et al., 2009). Coastal vulnerability is a matter of concern for many in India as about 35% of the population lives within 100 km of the country’s 7517 km long coastline. There is a need to study and understand the impact of sea level rise and coastal subsidence on these areas. Unfortunately, there are limited studies on the sensitivity of Indian coasts. Assessing this vulnerability is important for future planning and protecting coastal communities (Space Applications Centre, et.al, 2000).

According to projections by the Swaminathan Research Foundation (1981), sea levels are expected to rise by 16 cm and 32 cm by the years 2050 and 2100, respectively. A landmark study conducted by the Central Water Commission (CWC) in 2017 highlighted the vulnerability of 593 coastal districts, where 17% of the country’s population lives. The state of Tamil Nadu on the Eastern coast of India, which boasts the third longest coastline in the country, is at great risk. The state, which has 591 fishing villages along the coast, is projected to see an average sea level rise of 4.51 to 4.94 cm. In some districts like Tiruvallur, Chennai, and Kanyakumari, this rise is especially expected to reach 4.94 cm. In 2010, a research study highlighted the potential consequences of rising sea levels in Kanyakumari district, a coastal area spread over 13 sq. km (Roy et al., 2023). In addition to the immediate threat of flooding, the study highlighted a deeper risk of substantial land loss, which could result in the gradual submergence of coastal areas. This phenomenon is not limited to physical changes only; there is an anticipated change in coastal ecosystems, a process called coastal zonation change. The findings of this study emphasize the urgent need for the development and implementation of comprehensive climate change adaptation strategies and protection measures. Proactive efforts are needed to mitigate the potential impacts of challenges posed by rising sea levels on vulnerable Indian coastlines. By urgently addressing these concerns, there is an opportunity to safeguard not only the physical landscape but also the delicate balance of coastal ecosystems, highlighting the critical importance of sustainable practices and climate resilience in the face of environmental changes (Woodworth et al., 1999). NASA has introduced an online tool that shows the projected sea level rise, revealing that Kochi is expected to experience an increase of 0.11 m by 2030, 0.23 m by 2050, and 0.30 m by 2060. By 2130, the rise could reach 1 m. Kerala, a densely populated Indian state with a 590 km coastline, is at risk (TOI, 2021). The coastline is exposed to strong waves; rogue waves, known as “kallakadal”; and tsunamis, causing coastal erosion and habitat destruction. According to the Ministry of Environment and Forests of the Government of India, 63% of Kerala’s coastline is rapidly deteriorating. Kerala consists of nine coastal districts, including Kasaragod, Kannur, Kozhikode, Malappuram, Thrissur, Ernakulam, Alappuzha, Kollam, and Thiruvananthapuram. Of these, Thiruvananthapuram district is particularly vulnerable to erosion. The new tool highlights the urgency of adaptive measures and protection strategies in the face of threats from rising sea levels on India’s vulnerable coastlines (Roy et al., 2023). Of late, there has been increasing awareness of issues related to climate change, such as sea level rise and its associated risks. Major drivers of this increase include ocean warming, melting of mountain glaciers, and ice loss from ice sheets (IPCC, 2022). Climate change is a serious threat to India’s coastal regions, affecting millions of people who depend on the natural resources of these ecosystems. India’s coastline is highly vulnerable due to factors such as low-lying terrain, dense population, frequent cyclones, and environmental degradation. Sea level rise is a major concern, especially for the three megacities and many growing cities with large populations. The increasing frequency and intensity of cyclonic storms along with changing paths have put these coastal areas at greater risk. Floods caused by heavy rainfall often throw life into disarray in these cities (Nair, 2016). Sea level rose by 1.3 mm/year from 1901 to 1971, then by 1.9 mm/year from 1971 to 2006. Between 2006 and 2018, it increased even more to 3.7 mm/year, showing a concerning trend of rapid sea level rise (MOES, 2023).

When the sea level rises due to climate change and the changing rainfall patterns, it can make the soil too salty. This salty soil makes it difficult for farmers to grow crops (Schneider & Asch, 2020). In low-lying coastal areas and small islands, food production is limited and closely linked to climate change and rising sea levels. The impacts of sea level rise (SLR) are particularly pronounced in areas with dense coastal populations and well-established agricultural infrastructure. This not only disrupts the livelihood of local communities but also leads to loss of habitat for various species including birds, fish, and plants (Karim & Mimura, 2008). Sea level rise is primarily caused by climate change, caused by too many greenhouse gases in the air. When we burn fossil fuels and cut down trees, we release carbon dioxide, which warms the planet. As warming increases, ice from glaciers and ice caps melts, adding more water to the oceans. Additionally, warmer water occupies more space, causing sea levels to rise. Therefore, climate change is the main reason for rising seas (Burns, 2023). According to Dissanayake et al. (2009), India is considered at risk level 9 in the South Asia Disaster Report (SADR). Human activities are damaging the ecosystem and biodiversity on the Indian coast. This vulnerability happens when natural processes mix with human-induced coastal activities (Mujabar & Chandrasekar, 2013). The sensitivity of a coastline is influenced by a combination of natural factors, including its geology, shape, the interplay of currents and tides, variations in sea level, and the presence of tectonic activities and storm conditions in that specific area (Sudha Rani et al., 2015).

## Unveiling the impact: Rising sea levels and coastal consequences

The Ministry of Earth Sciences (MoES, 2023) shared a report on the impacts of climate change on India, particularly focusing on the impact on the Indian Ocean. The findings show that the tropical Indian Ocean has been warming in recent decades. Sea surface temperatures across the entire basin have increased by 0.15 °C per decade from 1951 to 2015, higher than the global average of 0.11 °C during the same period. Due to this warming trend, sea level in the Indian Ocean has risen at a rate of 1.06–1.75 mm per year from 1874 to 2004 and about 3.3 mm per year from 1993 to 2015. This recent rise is similar to the rise in global average sea level. A recent study warns that rising sea levels could pose a threat to two Indian cities, Chennai and Kolkata. The research shows that these cities, along with other cities such as Yangon, Bangkok, Ho Chi Minh City, and Manila, may face significant risks by 2100 due to climate change and higher greenhouse gas emissions (Bhavsar, 2023). The Sundarbans, located in West Bengal, presents a unique and complex scenario related to migration, climate change, and displacement, especially when compared to Odisha. The region has the distinction of being one of the most ecologically rich regions globally, hosting the world’s largest continuous mangrove forest spanning approximately 10,000 sq. km (UNESCO, 2023).

About 40% of this invaluable ecosystem lies within the borders of West Bengal, while the remainder extends into Bangladesh. However, despite its ecological importance, the Sundarbans faces formidable challenges. High levels of poverty and constant risks from natural hazards, including sea level rise, soil and water salinization, cyclones, and floods, collectively establish the region as one of the most dangerous areas of the Indian subcontinent. The looming threat of climate change has exacerbated these issues, threatening to worsen the already precarious situation in the Sundarbans. The delicate balance between environmental protection, socio-economic nutrition, and climate resilience underlines the complexity of the challenges facing this unique and important region (Panda, 2020).

Table 1 outlines the potential impacts associated with different scenarios of sea level rise ranging from 1 to 5 m. As sea levels rise, the severity of consequences, including flooding, infrastructure damage, and community displacement, also intensifies.

**Table 1:** Projected sea level rise (SLR) scenarios and impacts in India

SLR impact per meter	Impacted area (in sq. km)	Percentage (%)	Population impacted	Percentage (%)	Impacted GDP (in million USD)	Percentage (%)	Impacted agriculture extent (in sq. km)	Percentage (%)	Impacted urban extent (in sq. km)	Percentage (%)	Impacted wetland (in sq. km)	Percentage (%)
1 m	7811	0.24	4,446,570	0.44	16,223	0.59	2417	0.09	667	0.33	5606	1.30
2 m	13,562	0.42	7,447,115	0.74	27,414	0.99	4516	0.18	1114	0.55	9872	2.28
3 m	21,267	0.66	12,318,026	1.22	43,473	1.57	8226	0.33	1751	0.87	14,645	3.38
4 m	30,419	0.95	12,145,257	1.20	57,483	2.07	13,879	0.54	2622	1.30	19,513	4.51
5 m	39,520	1.23	24,626,888	2.44	72,263	2.61	19,973	0.78	3688	1.83	23,677	5.47

Source: Dasgupta, et al. (2009). The impact of sea level rise on developing countries: A comparative analysis

SLR threatens coastal areas, affecting population, wetlands, agriculture, gross domestic product, and urban areas. As sea levels rise, people living in these areas may have to relocate. Wetlands important for wildlife and protection from storms may be destroyed. Crops in fields in low-lying coastal areas may be ruined due to salt water. The economy, especially in sea-dependent coastal areas, may be affected by damaged infrastructure. Cities along the coast may face more problems like flooding and erosion, making it difficult to live there. It is important to act now to address these challenges. At 5 m of sea level rise (SLR), the impact on coastal areas becomes much stronger. The affected area spread over 39,520 sq. km (1.23%), affecting 24,626,888 people (2.44%). The economic consequences are evident with a GDP loss of USD 72,263 million (2.61%). The agricultural extent is 19,973 sq. km (0.78%) affected, while urban areas and wetlands are significantly impacted at 1.83% (3688 sq. km) and 5.47% (23,677 sq. km), respectively (Dasgupta, et al., 2009).

### Impact on coastal communities

Coastal communities may face problems like more salt in drinking water and damage to sewage systems due to rising sea levels. However, for Indigenous communities, who already deal with issues like limited access to safe water, sea level rise could make health problems even worse. This includes problems with food and water that cause diseases, impacts on mental health, and increased risk of chronic and infectious diseases and injuries. It also creates concerns about not having enough food and safe water. In short, while coastal areas will generally face problems, Indigenous communities may experience even greater health and well-being problems due to existing inequalities and the additional impact of rising seas (Berry and Schnitter, 2022). Rising sea levels pose a serious threat to coastal communities, putting people living in vulnerable areas such as fishing communities and marginalized groups at risk of losing their homes and livelihoods. This situation may create additional social, economic, and humanitarian challenges (Burns, 2023). As a case in point, in *Satabhaya*, a village in the Kendrapara district of Odisha, India, massive erosion caused by rising sea levels has created significant problems. To address these issues, the state launched a rehabilitation and resettlement program in 2011 for 571 affected families. Residents of coastal villages like *Satabhaya* have long faced the devastating effects of sea erosion. Many people in these erosion-prone areas have suffered significant losses of farmland and homes along the coast (Panda, 2020). Similarly, studies also reveal that the coastal communities in other parts of the country such as Mumbai, Chennai, Kolkata, Kochi, and Visakhapatnam suffer significantly due to rising sea levels. These cities face frequent flooding and erosion, leading to displacement and infrastructure damage. Economic activities like tourism, fishing, and shipping are disrupted, causing job losses and financial instability. Public health risks increase with waterborne diseases and contaminated water supplies. Additionally, environmental degradation and loss of cultural heritage further impact these vulnerable communities (Greenfield, 2023).

### Impact on livelihood

Sundarbans helps mankind with its natural resources by meeting the demand of people in diverse ways such as it provides shelter and provides sustainable livelihood to millions of people. It protects them from storms, cyclones, tidal surges, seawater seepage, and infiltration. The human-populated area is densely covered with forest. Sundarbans is losing its ecological diversity due to natural and anthropogenic reasons (Giri et al., 2008). Over the two decades leading up to 1985, the productivity of mangrove forests declined by 25% (Chaffey and Sandom, 1985). In West Bengal, Sundari trees are in danger of extinction due to past overharvesting for their valuable wood and the current threat of rising sea levels. An NCCR study shows that shoreline changes are caused by both natural factors and human activities. This shrinking coastline is not only causing loss of land and habitat but also affecting the livelihood of fishermen. They lose space to park boats, fix nets, and conduct fishing operations. Additionally, coastal flooding contributes to the decline in agriculture in India. Therefore, the combined effects of logging and sea level rise pose significant challenges to both the environment and communities dependent on coastal resources (MoEFCC, 2023). The impact of rising sea levels is being acutely felt by more than seven million coastal farming and fishing families. In particular, the most vulnerable areas include the Mumbai coast, the Kutch region, southern Kerala, and the Lakshadweep Islands on the west coast, as well as the deltas of the Ganges, Kaveri, Krishna, and Godavari on the east coast (Swaminathan Research Foundation, 1981).

### Coastal *erosion*

The 2023 report by the Ministry of Environment, Forest and Climate Change highlights a worrying issue: coastal erosion in India due to rising sea levels. This results in the loss of land, affecting valuable agricultural areas and coastal communities. The report shows that 33.6% of the Indian coastline is vulnerable to erosion, 26.9% is increasing, and 39.6% is stable. To address this, the findings have been shared with both central and state government agencies to implement shoreline protection measures. Between 1990 and 2016, India lost 235 sq. km of land due to coastal erosion. This loss threatens people’s livelihoods and homes, leading some to relocate voluntarily or, in extreme cases, with government intervention. Immediate action is vital to ensure the protection of these coastal areas and the safety of communities. This results in the most intense monsoon season in South Asia. Displacement is a significant concern, emphasizing the urgent need for measures to address the impact of these extreme weather events on communities (Panda, 2020). Data from satellites shows that the highest erosion is occurring in the coastal areas of West Bengal, with a significant 70% of the coastline affected. It is followed by the Kerala coast with a 65% erosion rate, Gujarat with 60%, and Odisha with 50%. In contrast, less than 50% of the coastlines of the remaining states are undergoing erosion, highlighting variation in the extent of this coastal challenge in different regions (Mohanty et al., 2017).

### Increased flooding

Rising sea level is causing problems in coastal areas. They flood low-lying areas, erosion of shorelines, cause coastal flooding, and allow salt water to seep into estuaries and nearby groundwater. This affects the lives and activities of people on the coast (EPA, 2021). Due to rising sea levels, there is a higher risk of floods in low-lying areas and river deltas in India. Cities such as Mumbai, Kolkata, and Chennai are more prone to frequent and severe floods, affecting millions of people and infrastructure. India is particularly sensitive to sea level rise, as the Indian Ocean is rapidly warming. Warming oceans, not just melting glaciers, contribute significantly to sea level rise. The impacts are already occurring along our coastline, emphasizing the urgent need to tackle these complex extreme events (TOI, 2019). Cyclones are becoming increasingly stronger due to warmer oceans, bringing more moisture and heat. This intensity increases flooding as storm surge increases sea levels. Cyclones in the region now bring more rainfall than ever before. Super Cyclone Amphan in 2020 caused widespread flooding, with salt water reaching tens of kilometers inland. This infiltration can harm farming for years. This is a serious issue, highlighted by Roxy Mathew Cole, a climate scientist at the Indian Institute of Tropical Meteorology, Pune, and author of the Intergovernmental Panel on Climate Change (Hindustan Times, 2023). Odisha is in a risky spot on India’s hazard map. Since the big cyclone in 1999, the state has focused a lot on managing disasters. By 2050, many people in Odisha’s coastal areas might face floods because of rising sea levels (The pioneer, 2019; Beura, D, 2015).

### Displacement and migration

The north-eastern seaboard is at risk due to its low elevation, which poses a risk of saltwater intrusion. Every year, farmland in the area gets ruined because it becomes too wet and salty for crops. Plants that can tolerate salt, like marsh plants, are spreading onto former farms, and this movement is called “marsh migration.” This is causing problems for farming and land in the region (Weissman et al., 2019). According to scientific studies and recent climate assessments of the Ministry of Earth Sciences, sea levels in the Indian Ocean are rising at an average rate of about 1.7 mm/year and at a faster rate of 3.3 mm/year in recent decades (1993–2015). This increase is forcing people in sensitive coastal areas to leave their homes and move to higher ground. This displacement causes social and economic problems for the affected communities (Greenfield, 2023). Between 2008 and 2018, about 3.6 million Indians were forced to relocate each year, mainly due to heavy monsoon rains causing floods (Panda, 2020).

### Saline intrusion

As sea levels rise, salt water may mix with freshwater sources such as rivers and groundwater. This affects the quality and availability of freshwater, causing problems for agriculture, drinking water, and ecosystems. When upwelling occurs along coastlines, salt water can come onto land, which is called saltwater intrusion. This happens during storms or high tides in low-lying areas, and also when salty water gets into the ground, making the water level under the soil go up (Weissman et al., 2019). Some parts of Gujarat, Tamil Nadu, and Andhra Pradesh are facing serious problem of seawater intrusion into the interior areas. This is happening because the groundwater level is lower than the average seawater level. From 2007 to 2017 the affected area has increased by approximately 500 sq. km and now reaches approximately 2600 sq. km. Chennai is the most affected among the affected cities, where seawater has entered up to a distance of about 14 km. Seawater intrusion threatens drinking water sources along India’s coast (Karekaat, 2020).

### Impact on biodiversity and ecology

Saline water can degrade water quality by releasing nutrients from fertilizers used in farms. Because of the way salt water interacts with soil, these nutrients can move around and end up through agricultural land into coastal water bodies such as creeks and marshes. Too many nutrients lead to excess algae growth. When the algae die, bacteria use the water’s oxygen to break them down. It can harm fish, animal homes, and the overall health of coastal ecosystems and wildlife (Weissman et al., 2019). India’s coastal ecosystems such as mangroves, coral reefs, and estuaries provide important habitats for diverse marine species. These ecosystems are threatened by rising sea levels. This leads to loss of biodiversity, decline in fisheries, and disruption of the natural balance of coastal ecosystems (Burns, 2023). Mangroves in the Indian Sundarbans are decreasing due to rising sea levels. The more sea levels rise, the more mangroves are destroyed. If conditions continue as they are, the Indian Sundarbans could lose between 42 and 80% of their mangrove areas by the end of the century (Samanta, et al., 2023). The coastal regions of India are home to diverse plants and animals, which provide important services to the environment. However, rising sea levels and greater coastal erosion are seriously threatening these ecosystems. This affects the plants and animals that depend on these areas. Critical habitats such as mangrove forests, coral reefs, and wetlands are particularly at risk. This puts various species at risk, destroying homes, reducing fish catches, and disrupting marine ecosystems. This is a big problem for the balance of nature on the coast (Greenfield, 2023). Even modest sea level rises can have harmful effects, destroying coastal habitats and expanding inland, flooding wetlands, aquifers, and salt-contaminated agricultural soils. The interconnectedness of these ecosystems makes them highly vulnerable to the consequences of sea level rise, highlighting the urgent need for sustainable measures to reduce impacts on food production and biodiversity in these vulnerable areas (Karim & Mimura, 2008). Sea level rise threatens biodiversity in India, known for its diverse maritime life, ranging from small seahorses to giant whale sharks. Coastal habitats, essential for many species, including 26 types of freshwater turtles and five species of sea turtles, are at risk. For example, Odisha witnesses over 200,000 olive ridley turtles nesting annually. As sea levels increase, these crucial habitats face loss, endangering these species and disturbing the balance of marine ecosystems (Roy et al., 2023).

### Impact on infrastructure and tourism

Tourism depends on the economic activities of the people. If sea level rise causes damage to places and people become aware of it, it could have a serious impact on tourism. The loss of these economic activities will have a direct impact on the prosperity of tourism in those coastal areas (Yong, 2021). Rising sea levels threaten key infrastructure along India’s coastline, including ports, airports, power plants, and roads. Damage or disruption to infrastructure could result in economic losses, hinder development, and affect coastal industries such as tourism and shipping (Burns, 2023). Higher sea levels are a big problem for tourism in the Bahamas islands of New Providence and Paradise Island. If the sea goes up by 1 m, it could flood lots of tourist places in stormy areas. Rising seas are not the only issue—when you add storm surges (ranging from mild to severe), a large portion of hotels and resorts, about 34 to 83%, could be at risk of flooding. This could cause serious damage to the tourism industry, affecting many hotels and resorts (Pathak, et al., 2021). The report by Byravan et al. (2010) indicates that rising sea levels significantly threaten Tamil Nadu’s coastal infrastructure. The projected replacement cost for damaged ports, power plants, and major roads due to sea level rise is estimated to be between $10 and 12 billion, based on 2010 values.

### Water security and food production

Rising sea levels could harm farming and food security in coastal areas. Excess salt and flooding can damage agricultural land, reducing crop growth and water available for crops. This makes it difficult to produce enough food and harms the livelihoods of people dependent on farming (Turral, et al., 2011). Sea level rise could lead to the loss of cultivable land and freshwater resources, affecting coastal biodiversity and food security. It is essential to address these challenges through sustainable development practices and measures to mitigate the impact of climate change on India’s coastal environment (Nair, 2016). When sea levels rise and rainfall patterns change, it can cause the soil to become too salty. This salty soil makes it difficult for farmers to grow crops, which means less food for everyone. Furthermore, because the soil is affected, it can lead to a shortage of water for people’s use. Therefore, changes in sea level and precipitation may make it harder to grow enough food and get enough water for everyone (Schneider & Asch, 2020).

## Strategies for resilience: Adapting to sea level rise

In 1991, the Government of India established the Coastal Regulation Zone (CRZ) following the Environment Protection Act of 1986. This rule covers coastal areas affected by low tidal activity up to 500 m from the high tide line, including seas, bays, estuaries, gulfs, rivers, and backwaters. In 2011, the Ministry of Environment, Forest and Climate Change introduced new coastal rules. In 2019, the special provisions for Goa and Kerala were removed, and a similar adjustment was implemented in all coastal states (Roy et al., 2023).

The Government of India is actively addressing sea erosion and coastal protection through the Coastal Regulation Zone Notification, 2019. It is issued by the Ministry of Environment, Forest and Climate Change; this notification allows erosion control measures and establishes no development zones (NDZ) in various coastal areas. The goal is to conserve coastal stretches and marine areas and ensure livelihood security for local communities while preventing encroachment and erosion. Monitoring and enforcing these rules is important for effective security. Community participation and awareness are also important for successful coastal management (MoEFCC, 2019). The National Center for Coastal Research (NCCR), part of the Ministry of Earth Sciences, has been tracking changes in India’s coastline for 28 years (1990–2018). It is observed that 33.6% of the coastline is vulnerable to erosion, 26.9% is growing (accretion), and 39.6% is stable. This information helps to understand and manage coastal vulnerabilities over time (MoES, 2022).

In response to the super cyclone in 1999, the Odisha government launched efforts to rehabilitate the people of Satabhaya, leading to the Satabhaya Resettlement and Rehabilitation Yojana in 2011. This program successfully relocated 571 families to Bagapatia, 12 km away. Although the land allotment was based on outdated surveys and did not account for newer generations, this effort marked the relocation of India’s first climate refugees. The initiative provided them with new homes, agricultural land, and facilities, offering a crucial response to the impacts of sea level rise and coastal changes (Panda, 2020).

### Suggestion for addressing the threats

The Sundarbans play a vital role in supporting the livelihoods of more than 1.3 million people, contributing significantly to the well-being of the wider population of 4.4 million inhabitants. Beyond its socio-economic importance, this mangrove forest acts as a natural buffer, reducing the physical impacts of cyclones in the region. A notable example is the moderate impact seen during Cyclone Amphan, where the presence of the Sundarbans significantly reduced the severity of impacts in Bangladesh (Panda, 2020). Drawing from the findings of the studies on shoreline changes, it is recommended to implement measures addressing sea level rise threats in coastal areas. These measures could include the following strategies:

#### Measures to address SLR

India should invest in protecting its coasts by building sea walls, dams, and afforestation. These steps will help reduce the risks of erosion and flooding, especially in areas that are more vulnerable.

#### Climate resilience

To deal with the long-term effects of rising sea levels, it is important for India and other countries to work together to reduce the amount of greenhouse gases in the air. This can be done by using more renewable energy, improving energy efficiency, and adopting sustainable practices globally.

#### Sustainable coastal management

India needs to plan how it uses its coastal areas by balancing development with protecting the environment. This includes getting input from the people who live there, monitoring how the coast is changing, and ensuring that land use practices are sustainable.

#### Empowering coastal communities

People living in coastal areas must be given the tools and knowledge to cope with rising sea levels. This includes involving them in decisions about their communities, preparing for disasters, and creating strategies to adapt to changes.

#### Global cooperation

India should actively work with other countries in the global discussion on climate change like the United Nations Framework Convention on Climate Change. By doing so, India can seek shared responsibilities and support from the international community to help put in place effective plans to tackle rising sea levels. This collaboration at a global level is important to tackle the wider impacts of this issue.

## Conclusion

Climate change–driven sea level rise is a pressing global issue, exacerbated by human activities such as greenhouse gas emissions. This leads to Earth warming, melting polar ice, and seawater expansion. The consequences include heightened coastal erosion, increased flooding, and threats to communities and ecosystems. Addressing this urgent challenge requires international cooperation, sustainable practices, and resilient infrastructure. It is not just an environmental imperative but a societal responsibility to safeguard our planet and future generations. India, facing the threat of rising sea levels, needs a multi-faceted strategy. This involves adapting to changes, mitigating climate impacts, engaging communities, and fostering global collaboration. Proactive steps and investments in sustainable development can help minimize risks. Non-Governmental Organizations (NGOs) play a pivotal role in driving impactful change. Through concerted efforts, India can contribute to global solutions, emphasizing the importance of collective action in securing the well-being of coastal communities and the health of our planet.

To tackle the challenges of rising sea levels in India, global cooperation and community involvement are crucial. The relocation of *Satabhaya’s* climate refugees shows that effective solutions require both international support and local engagement. Concerted interventions transcending geographic boundaries and working together with affected communities would help develop better strategies to protect people, preserve ecosystems, and adapt to climate change. Leveraging global expertise and combining it with local action is key to addressing these pressing issues.

## Data Availability

The data used in this study is publicly available and has been cited within the text, with references provided. The datasets of Global Sea Level rise generated and analyzed from the study of Church, J. A., and N. J. White (Church and White, 2011), Sea-Level Rise from the Late 19th to the Early 21st Century, Surveys in Geophysics, 32(4-5), 585-602, doi: http://dx.doi.org/10.1007/s10712-011-9119-1. The datasets in Table.1 Projected Sea Level Rise (SLR) Scenarios and Impacts in India generated and analyzed from the study of Dasgupta et al. (2009).The impact of sea level rise on developing countries: a comparative analysis (English). Policy, Research working paper; no. WPS 4136 Washington, D.C.: https://doi.org/10.1007/s10584-008-9499-5 World Bank Group. http://documents.worldbank.org/curated/en/156401468136816684/The-impact-of-sea-level-rise-on-developing-countries-a-comparative-analysis
